# Assessment of Attitudes and Intentions towards COVID-19 Vaccines and Associated Factors among General Populations of Pakistan: A Cross-Sectional Study

**DOI:** 10.3390/vaccines10101583

**Published:** 2022-09-21

**Authors:** Saadullah Khattak, Muhammad Idrees, Hafiza Iqra Iqbal, Maqbool Khan, Nasir Assad, Muhammad Naeem Khan, Muhammad Tufail Yousaf, Muhammad Farooq, Chang-Yong Yang, Dong-Dong Wu, Xin-Ying Ji

**Affiliations:** 1 Henan International Joint Laboratory for Nuclear Protein Regulation, School of Basic Medical Sciences, Henan University, Kaifeng 475004, China; 2 School of Life Sciences, Henan University, Kaifeng 475004, China; 3 Primary and Secondary Health Care Department, Lahore 54000, Pakistan; 4 Sino-Pak Center for Artificial Intelligence, Pak-Austria Fachhochschule: Institute of Applied Sciences and Technology, Haripur 22620, Pakistan; 5 Institute of Chemistry, University of Sargodha, Sargodha 40100, Pakistan; 6 School of Social and Behavioral Sciences, Nanjing University, Nanjing 210023, China; 7 Institute of Microbiology, Faculty of Veterinary and Animal Sciences, Gomal University, Dera Ismail Khan 29111, Pakistan; 8 Department of Microbiology, University of Swabi, Swabi 23430, Pakistan; 9 School of Nursing and Health, Henan University, Kaifeng 475004, China; 10 School of Stomatology, Henan University, Kaifeng 475004, China

**Keywords:** COVID-19, vaccines, attitudes, intentions, Pakistan

## Abstract

Objective: The goal of public health in combatting COVID-19 is to increase herd immunity. However, vaccine reluctance makes attaining herd immunity a worldwide challenge. This investigation aimed to identify negative and positive attitudes and intentions about COVID-19 vaccinations. Methods: A cross-sectional online survey was conducted once free COVID-19 vaccines became available in Pakistan in 2021. 4392 Pakistanis aged 18 and older were surveyed from seven administrative units between 1 July and 30 August 2021. Online structured questionnaires were utilized to collect data using a simple sampling procedure. The questionnaires were divided into three major sections: sociodemographic, health factors, and attitudes toward COVID-19. Results: The survey link was shared with approximately 4500 participants. 97.6%(4392) completed the survey once begun. Frequency, percentage and Chi-square tests were used to analyze statistical data. Most of the participants in the research were men (2703 (61.54%)), 3277 (74.61%) were aged 18–29 years, and 1824 (41.53%) were residents of the Khyber Pakhtunkhwa province. (18.69%) Respondents expressed COVID-19 vaccine hesitancy, whereas 36.66% of participants liked getting the Sinopharm and Sinovac vaccines and (35.84%) of participants preferred the Pfizer vaccine. A significant number of participants (38.05%) were concerned about the vaccine’s unexpected side effects Thus, it is essential to realize that many participants were concerned about the vaccine’s unexpected side effects. Conclusions: The overall high level of concern about the unforeseen side effects of COVID-19 vaccines, as well as widespread vaccine hesitancy among Pakistani populations and its predictors, should be taken into account if public health intervention campaigns in Pakistan are changing negative attitudes and improving compliance with regard to COVID-19 vaccines.

## 1. Introduction

Currently, the Coronavirus disease 2019 (COVID-19) poses a healthcare challenge. COVID-19 is spread quickly by aerosols from coughing or sneezing, nasal discharge, saliva, urine, faeces, and close contact with an infected person [1,2]. Infection with COVID-19 can be asymptomatic, which may expedite its spread [3,4,5].

Vaccination has been necessary to minimize disease, disability, and mortality [6]. Vaccines are estimated to save the lives of four to five million people every year from deadly infections [5]. They were utilizing this advantage efficiently, resulting in countless outstanding achievements in the fight against influenza, polio, diphtheria, tetanus, hepatitis B, MMR (Mumps, Measles and Rubella) and pertussis. Yet, there is a vaccine coverage disparity among nations, including within countries [7]. Vaccine hesitancy was identified as one of the most significant threats to world health in 2019 by the World Health Organization (WHO) [8]. Vaccine hesitancy leads to measles, diphtheria, and pertussis epidemics due to incorrect vaccine safety concerns [6]. Religious and political leadership can influence vaccine rejection [9]. Because of religious convictions, Thailand, Vietnam and Mongolia have a significant aversion to vaccination [10]. Furthermore, unique ideas, such as whether active immunity from natural infection is preferable to vaccination or if a healthy lifestyle might inhibit disease development, play a role [11].

The immunization efforts of the Pakistani population are far from meeting the global vaccination targets. BCG (Bacillus Calmette Guerin) vaccination coverage is 80%, polio vaccination coverage is 60%, and measles vaccination coverage is 67% [12]. Vaccination product promotions tend to be influenced by political, religious, and commercial factors, as well as logistical challenges, inadequately trained health care professionals, a lack of parental awareness and education, as well as factors related to social norms, political systems, and the environment [12,13]. Previous vaccination campaigns had relied heavily on religious and political figures promoting conspiracy theories. These beliefs have also contributed to Pakistan’s failure to eradicate polio [14]. Essential features for COVID-19 immunization uptake include government clearance of its safety and efficacy, employer endorsements, and expenditures [14]. Parents are also suspicious of the innovative COVID-19 vaccination [15]. The vaccine has been actively opposed by anti-vaccination groups, who attempt to deny the existence of COVID-19, in particular [16]. The COVID-19 vaccination program in Pakistan is managed by the National Command and Control Center (NCOC); frontline healthcare workers who are involved with the care and treatment of patients who have confirmed or suspected COVID-19 are the first to be vaccinated, followed by other healthcare workers and the general population [17].

The acceptance of the COVID-19 vaccine varies from country to country. Approximately half of Americans in the United States agree with vaccination [18]. In Indonesia, 93.3% support it [19], 62% approve of it in France, Denmark has an 80% support rate [17], and 59% of Italians [20], want to get vaccinated. When the COVID-19 vaccine becomes available in Pakistan, inaccurate narratives and religious conspiracy theories may influence people’s decisions to take the vaccine [21,22]. In consideration of the COVID-19 pandemic, there is an immediate requirement for more current information on public attitudes toward COVID-19 vaccines and related variables to adapt suitable activities. The current study intends to assess the frequency of attitudes and intentions concerning COVID-19 vaccination and to identify drivers of unfavourable attitudes and intentions. Investigating determinants of intentions toward the COVID-19 vaccination might assist authorities and governments in identifying and implementing relevant actions to alleviate concerns and reluctance and boost trust in the vaccine.

## 2. Subject and Methods

### 2.1. Participants and Study Design

The online survey was developed from 1 July to 30 August 2021 via a link to Google Forms (https://docs.google.com/forms/, accessed on 1 September 2021, Survey, Supplementary file S1). The survey’s data collection included the general population with only one age limit, participants had to be at least 18 years of age; there were no further exclusion criteria. The survey link was shared with approximately 4500 participants, and 97.6% of them (4392) completed the survey once begun. Pakistan’s estimated population on 1 July 2021 was 225,199,937 according to the World Population Review [23]. The Raosoft sample size calculator was used to calculate the needed sample size [24]. A sample size of 385 was calculated. For the sample estimate, a 50% response distribution was assumed, a 5% margin of error was taken, and a 95% confidence level was used. Several channels were sent the poll URL after a call for participants. WeChat, LinkedIn, Facebook, Messenger, and Instagram were also used to increase general public interaction. The survey took about five minutes to complete. Core members could only access the repository data, ensuring privacy.

### 2.2. Variables and Questionnaire Instruments

A structured survey was prepared after reviewing the relevant scientific literature and available resources on COVID-19 [19,25,26,27,28,29,30]. This survey was written in English, the preferred language of higher education and the national language of Pakistan. The utilized questionnaire consists of three parts: (1) sociodemographic information and (2) general public attitudes toward COVID-19 vaccinations, and (3) intentions for COVID-19 vaccinations.

### 2.3. Information about Socio-Demographics

Participants’ gender, age, administrative unit, residence, marital status, chronic diseases, employment status, and education were collected under the “sociodemographic characteristics” section of the survey. The participants’ educational level was classified as high school, college, graduate, or post-graduate. Occupations included full-time, part-time, unemployed, housewives, retired, and workers.

### 2.4. The Attitude towards COVID-19 Vaccines

The six questions in this section were about attitudes toward COVID-19 vaccines, such as “Have you had a flu vaccination?”; “Has any family member had COVID-19?”; and “Does the ministry of health provide adequate information about COVID-19 vaccines?”; Intentions to receive the COVID-19 vaccine; COVID-19 vaccine preferences; and “I do not believe the vaccine works”.

### 2.5. The Intentions toward COVID-19 Vaccines

Fourteen questions were asked of the participants to assess their intentions toward the COVID-19 vaccinations, which are scored on a five-point Likert scale, such as strongly disagree, disagree, neutral, agree and highly agree.

### 2.6. Operational Definitions

Positive Value: Research participants answer positively to 50% or more of the attitude and opinion questions.

Negative Value: Less than 50% of attitude and intentions questions are answered by research participants.

### 2.7. Dependent Variable

Attitude and intentions of general Pakistani population toward COVID-19 vaccine acceptance.

### 2.8. Independent Variable

Sociodemographic features include gender, age, administrative units, residence, marital status, chronic disease, employment status, and education.

### 2.9. Statistical Analysis

To conduct a descriptive and statistical analysis of the responses obtained from Google Forms, the results were converted into Microsoft Excel format and then exported to IBM SPSSv20. Statistical Analyses were performed in Jupyter Notebook using Python 3 with Pandas, Matplotlib and stats libraries. The demographic characteristics of the research participants were displayed using descriptive medical statistics such as frequency and percentage. Chi-square tests examined the association between intentions and sociodemographic variables, which are also used in epidemiological, public health, and medical research. A *p*-value of 0.05 was used to determine statistical significance.

### 2.10. Consent to Participate

Our survey’s first page consists of an informed consent page that describes the investigation of respondents. To continue the study, participants had to consent (or decline) participation, and there was no incentive for individuals to participate in this voluntary survey.

### 2.11. Ethical Approval

The Department of Microbiology approved the study Ethical Research Committee at University of Swabi in Pakistan (study registration no: 1(3)-Micro-8/UoS/2022/1674).

### 2.12. Results

#### Information about Socio-Demographics

**Figure 1:**
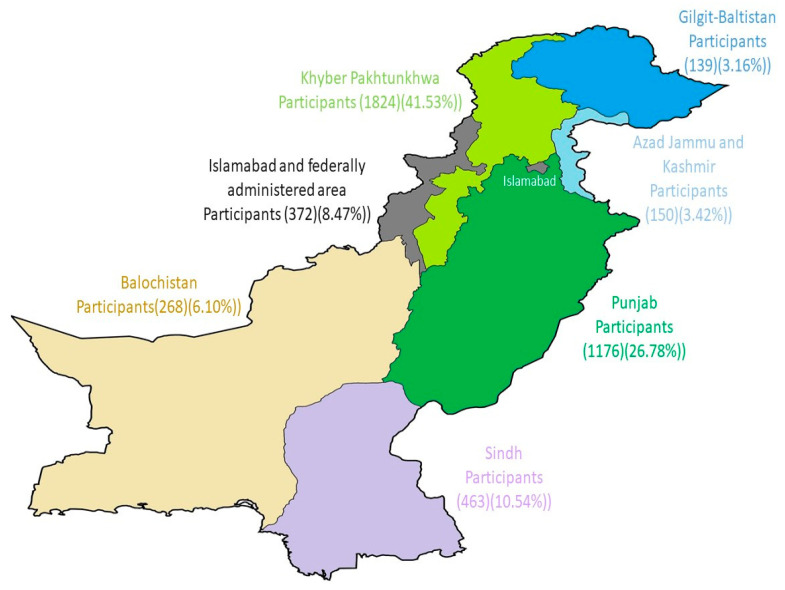
Distribution of participants according to the area.

Most of the research participants were males, (2703 (61.54%) and 3277 (74.61%) were aged 18–29 years; 1824 (41.53%) were residents of the Khyber Pakhtunkhwa province (Figure 1). About 1043 (23.75%) were married and 3349 (76.25%) were single. More than half, 2830 (64.44%), were from urban areas. Among the study participants, the majority (80.76%) had no chronic diseases, 174 (3.96%) did not have any medication hypersensitivity, and 206 (4.69%) had cardiac disease. More than half, 2994 (68.17%), were students, and 850 (19.35%) were full-time workers. The majority of the 1718 (39.12%) were university graduates, 1119 (25.48%) were postgraduates, 1537 (34.99%) were from college, and 18 (0.41%) from school, respectively. Table 1 illustrates a detailed analysis of the demographics of the study participants.

## 3. The Attitude towards COVID-19 Vaccines

**Figure 2:**
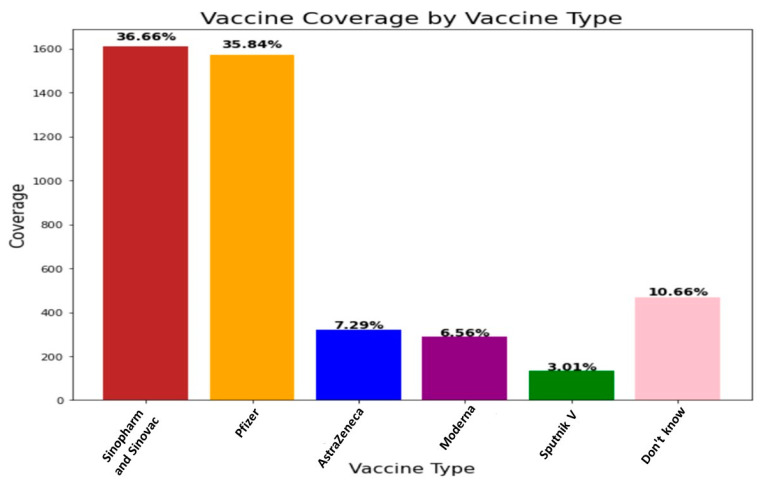
Participant’s response to Vaccine types.

**Figure 3:**
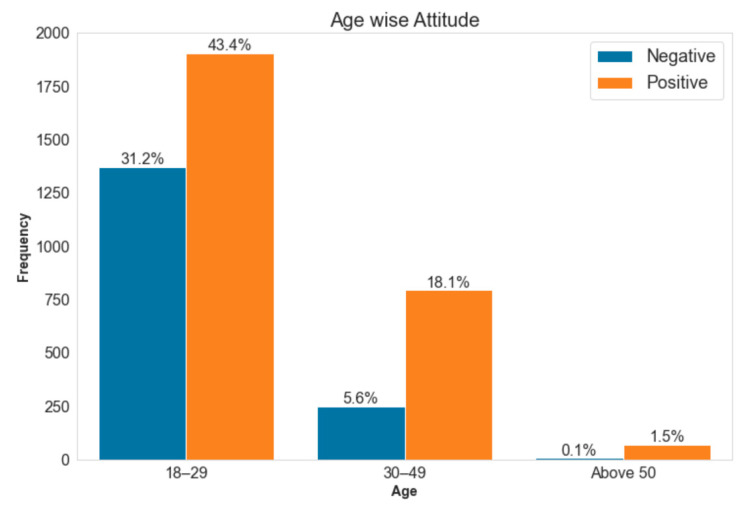
Association between attitude and age. Note: Here percentage is calculated based on a total number of observations, i.e., 43.4% of the total participant’s attitude is positive. In Table 3, the row-wise percentage is calculated.

Approximately 3102 participants (70.62%) had never taken the flu vaccination. At the same time, 37.4% of respondents had a family member who had COVID-19. Most research participants believed that the Ministry of Health provided sufficient information on COVID-19 vaccines. Concerning intent to get the COVID19 vaccination, 18.69% were hesitant, 4.92% were unwilling, and just 76.39% were willing to do so. About 36.66% and 35.84% of survey participants preferred the Sinopharm, Sinovac and Pfizer vaccines, respectively. Figure 2 and Figure 3, equating to 6.55%, 3.01%, and 7.29% who preferred the Moderna vaccine, Sputnik V vaccine and AstraZeneca vaccine, respectively (Table 2 and Table 3).

## 4. The Intentions toward COVID-19 Vaccines

**Figure 4:**
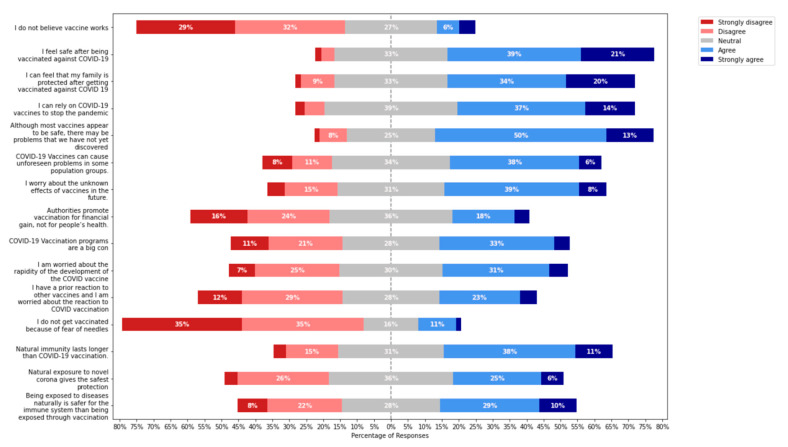
The Intentions toward COVID-19 vaccines among the general populations (*n* = 4392).

The most generally stated reason for vaccine acceptance was to defend oneself from obtaining the virus (1731 (39.41%)), followed by 1534 participants (34.92%) who feel sure that their family is safe after being vaccinated against COVID-19. 1654 (37.66%) participants agree that COVID-19 vaccines can end the pandemic. Half of the participants, 2250 (50.66%), believe that although most vaccinations seem to be safe, there may be issues that haven’t yet been identified; COVID-19 vaccines might create unforeseen problems among different demographic groups according to 38.05% of respondents, and 1747 (39.78%) people were concerned about the unknown future effects of vaccinations. More than 1064 people (24.23%) disagreed that the government promotes vaccination for financial benefit instead of for people’s health. In comparison, 1488 participants (33.88%) believed that COVID-19 vaccination schemes were a big con. Approximately 1384 (31.51%) participants were concerned about the rapidity with which the COVID vaccine is being produced, and 1044 (23.77%) had an early reaction to prior vaccinations and were worried about the reaction to COVID-19 vaccination. Just 490 participants (11.6%) said that they do not get vaccinated due to their fears about needles. Approximately 1698 participants (38.65%) indicated that natural immunity lasted longer than COVID-19 vaccination, 1140 participants (25.96%) agreed that natural exposure to the novel Coronavirus provides the safest and most effective protection, and 1288 participants (29.33%) believed that natural disease contact is a better method of preventing disease than vaccination (Table 4 and Table 5, and Figure 4).

## 5. Discussion

Vaccination is one of the twenty-first century’s most important public health breakthroughs. Vaccination fear represents one of the most critical public health challenges today. The significant degree of vaccination apprehension reveals that the obtainability of COVID-19 vaccines is not the source of concern.

Prior published studies indicated that proper public knowledge is a significant predictor of disease prevention and control. Compared to the more severe second wave, the Pakistani individuals and cancer patients surveyed during the first wave of COVID-19 infection were aware of COVID-19 prevention measures [31,32]. More than half of the participants (38.05%) expressed a high level of fear about the vaccine’s unforeseen effects, which is consistent with a US study which revealed that the most common negative attitude toward COVID-19 vaccines was significant worry about its unforeseen effects [33]. Furthermore, according to Chinese research, 48% of participants postponed vaccination until vaccine effectiveness was proven [34]. Several scientists have expressed concern about the safety of vaccinations. The rapid production rate of vaccines and the doubts raised by some scientists and health experts may generate suspicion about the COVID-19 vaccine [35]. In the study, 39.78% of participants expressed concerns about possible adverse effects when the vaccine first became available. In a similar study, Pogue et al. found that 63% of participants were concerned about the side effects of COVID-19 vaccination [36].

A 2021 study that looked at global trends in COVID-19 vaccination reluctance revealed that men, older people, and those who had previously had influenza vaccines were less prone to hesitancy, which confirmed our results [37]. They also observed that those who lived in cities and those in the upper or middle tiers considered affluent were less inclined to be fearful of vaccines. Regardless, our study found no evidence of a link between these factors [37]. Our results indicated that 76.37% of participants were highly likely to receive a COVID-19 vaccination, which was much lower than the reported rates in Brazil (89%), Italy (81%), and the United Kingdom (80%), but was higher than the reported rates in Canada (71%), the United States (57%), Turkey, and France (49% for both) [37]. The following elements were discovered to have a more extensive effect on vaccination ratios and might be utilized to develop public health strategies and communications to boost vaccination proportions. Knowing that the COVID-19 vaccine is both efficient and safe will increase a considerable percentage of the trial group to vaccinate. These two variables are part of the VCI and are currently investigated in a major worldwide investigation [38]. They are expected to have the greatest effect on COVID-19 vaccination acceptance. Instilling trust in vaccines and the companies that supply them will be critical if proper, honest reporting of the risks and benefits ratios is provided on social media and news platforms [39]. Although confidence in vaccine manufacturers has been recognized as a major factor in vaccination acceptance, this must be maintained by focusing on the stringent regulatory requirements that the companies must follow, which can be communicated through uniform and transparent healthcare communication. Participants’ desire to vaccinate was also influenced by their belief that the COVID-19 vaccination would protect individuals surrounding them. Though vaccinated individuals can also transmit SARS-CoV-2, transmission is lowered, which ensures that friends and family are kept secure [40]. Even though confidence in vaccine companies has been established as a crucial determinant in vaccination acceptability [35], this has to be maintained by concentrating on the strict regulatory criteria that organizations must follow, which may be communicated through clear and uniform healthcare communication. Healthcare experts will use continuously updated connectivity to correct info on the COVID-19 vaccine. This will help battle non-evidence-based anti-vaccination propaganda by detailing benefits and concerns, assessing new data as it arises, and customizing it to people seeking treatment [41,42].

The public health authorities must offer precise, simple-to-understand data on the SARS-CoV-2 virus. Vaccinations to the public at large, such as those previously stated in the H1N1 pandemic [43]. This will help to reduce the confusion caused by disinformation in the media and on the internet. Moreover, we acknowledge Seale et al.’s recommendations [42], which include adjusting messaging and incorporating local leaders in communicating vaccination knowledge among minority ethnic groups, given the recognised effect of comparable social groups and community leaders. Participation and training are critical for healthcare staff, as they play a crucial role in demonstrating health-promoting behaviour for the public [44]. Several healthcare professionals in Australia have previously received the mandated influenza vaccine and compulsory COVID-19 vaccination for elderly care employees and healthcare staff in all regions and states [45].

Additionally, experience suggests that most people employed in other health institutions have been vaccinated, with just a tiny percentage refusing the COVID-19 vaccination since the required strategy was changed. Health providers’ ideas and attitudes against COVID-19 vaccination may mirror similar community concerns reported in the UK, with reluctance being more common in non-white British healthcare professionals, female sex, and those of younger age [46]. Given the risks to patients and staff and the need for booster (or third dose) vaccines in the present outbreak, it is crucial to comprehend the legislation, information, attitudes, and opinions in influencing this behaviour.

In order to increase the number of COVID-19 vaccinations, it is important to reach people where they are with persuasive information from reliable sources. Optimizing or modifying the individual vaccination plan through the dynamic monitoring of vaccination efficacy is one potential solution. Volunteers and staff can go to areas where vaccination rates are low to answer people’s questions about vaccinations and set up appointments for vaccinations for themselves and their loved ones. The elderly, those with disabilities, those who are confined to their homes, and residents of rural areas may be the most vulnerable groups in the community. For those who struggle to get to a vaccination site, at-home vaccinations might be a good option. In cooperation with nearby pharmacies, paramedics and healthcare professionals provide vaccinations door-to-door. Residents can fill-out an online form or make a phone request for at-home vaccinations. It is essential to use reliable channels of communication to inform communities about COVID-19 vaccines and vaccination locations. Gaining the confidence of their communities is essential for health departments if they want to see an increase in demand for COVID-19 vaccinations [47,48,49,50,51,52,53].

This research has certain limitations. It only included people who had access to the internet, eliminating the most marginalized communities. Among the respondents, only about 1% were older than 50 years of age, and about 40% were graduates. Furthermore, the data’s cross-sectional character restricts our capability to make inferences regarding direct relationships. The sample, on the other hand, is representative of the Pakistani population, and the use of mixed approaches allowed for a better understanding of the results as well as the order of the ideas.

## 6. Conclusions

Despite the overall mortality, hospital readmissions, and death caused by COVID-19, the results of this study on vaccine hesitancy are noteworthy given the documented safety and efficacy of widely tested vaccinations to prevent these problems and the urgent need to accelerate immunization internationally, particularly in Pakistan. Vaccine intentions are influenced by vaccination efficacy, effectiveness, trust in corporations, and doctor advice. Further studies must be conducted on vaccination reluctance among specific groups, such as healthcare professionals and linguistically and culturally diverse individuals. COVID-19 vaccine uptake could be improved with this research, especially in Pakistan with the Delta variant. There is a high level of concern about unforeseen side effects of COVID-19 vaccines, as well as widespread hesitancy among Pakistani populations and its predictors when implementing public health intervention campaigns to change negative attitudes and improve uptake of COVID-19 vaccines.

## Figures and Tables

**Table 1:** Demographic characteristics of the study population (*n* = 4392).

S.No.	Variable		Frequency	Percentage (%)
1	Gender	Male	2703	61.54
		Female	1683	38.32
		I prefer not to say	6	0.14
2	Age	18–29	3277	74.61
		30–49	1041	23.70
		Above 50	74	1.69
3	Administrative Units	Khyber Pakhtunkhwa	1824	41.53
		Punjab	1176	26.78
		Sindh	463	10.54
		Islamabad Capital Territory	372	8.47
		Baluchistan	268	6.10
		Azad Jammu and Kashmir	150	3.42
		Gilgit-Baltistan	139	3.16
4	Residence	Urban	2830	64.44
		Rural	1562	35.56
5	Marital status	Single	3349	76.25
		Married	1043	23.75
6	Chronic disease	No chronic disease	3547	80.76
		Cardiac disease	206	4.69
		Hypertension	174	3.96
		Overweight/obesity	168	3.83
		Others	132	3.00
		Asthma or respiratory disease	108	2.46
		Kidney or liver disease	51	1.16
		Cancer	6	0.14
7	Employment Status	Student	2994	68.17
		Full-Time	850	19.35
		Part-Time	180	4.10
		Unemployed	162	3.69
		Housewife	114	2.60
		Retired	74	1.68
		Worker	18	0.41
8	Education	Graduate	1718	39.12
		College	1537	34.99
		Postgraduate	1119	25.48
		High School	18	0.41

**Table 2:** The attitude toward COVID-19 vaccines among the general population (*n* = 4392).

Q.No	Questions/Variables	Values	Frequency	Percentage
1	Have you had a flu vaccination?	Never	3102	70.62
		Yes last year	486	11.07
		Yes (a long time ago)	450	10.25
		Yes this year	282	6.42
		Yes every year	72	1.64
		No	2337	53.22
		Yes	1851	42.14
		Don’t know	204	4.64
2	Has any family member had COVID-19?	Yes	2219	50.52
		No	2083	47.43
		Don’t know	90	2.05
3	Does the ministry of health provide adequate information about COVID-19 vaccines?	Yes	3365	76.62
		No	541	12.32
		Don’t know	486	11.06
4	Intentions to receive the COVID-19 vaccine	willing to take the vaccine	3555	57.39
		hesitated to take the vaccine	821	18.69
		not willing to take the vaccine	216	4.92
5	COVID-19 vaccine preferences	Sinopharm and Sinovac vaccine	1610	36.66
		Pfizer vaccine	1574	35.84
		I don’t have enough information to decide	468	10.65
		AstraZeneca	320	7.29
		Moderna vaccine	288	6.55
		Sputnik V vaccine	132	3.01
6	I do not believe the vaccine works	Strongly disagree	1275	29.02
		Disagree	1425	32.45
		Neutral	1194	27.19
		Agree	288	6.56
		Strongly agree	210	4.78

**Table 3:** Association between Attitude and demographic variables (*n* = 4392).

S.No.	Variable	Categories	Positive Frequency (%age)	Negative Frequency (%age)	Chi-Square	Significance (*p*-Value)
1	Gender	Male	1863 (68.92)	840 (31.08)	112.40	0.000
		Female	905 (53.77)	778 (46.23)		
		I prefer not to say	0 (0)	6 (100)		
2	Age	18–29	1906 (58.16)	1371 (41.84)	138.10	0.000
		30–49	794 (76.27)	247 (23.73)		
		Above 50	68 (91.89)	6 (8.11)		
3	Administrative Units	Khyber Pakhtunkhwa	1038 (56.91)	786 (43.09)	505.98	0.000
		Punjab	654 (55.61)	522 (44.39)		
		Sindh	463 (100)	0 (0)		
		Islamabad Capital Territory	282 (75.81)	90 (24.19)		
		Baluchistan	111 (41.42)	157 (58.58)		
		Azad Jammu and Kashmir	150 (100)	0 (0)		
		Gilgit-Baltistan	70 (50.36)	69 (49.64)		
4	Residence	Urban	1641 (57.99)		86.66	0.000
		Rural	1127 (72.15)	435 (27.85)		
5	Marital status	Single	2074 (61.93)	1275 (38.07)	7.25	0.0071
		Married	694 (66.54)	349 (33.46)		
6	Chronic disease	No chronic disease	2118 (59.71)	1429 (40.29)	410.78	0.000
		Cardiac disease	206 (100)	0 (0)		
		Hypertension	174 (100)	0 (0)		
		Overweight/obesity	36 (21.43)	132 (78.57)		
		Others	102 (77.27)	30(22.73)		
		Asthma or respiratory disease	96 (88.89)	12 (11.11)		
		Kidney or liver disease	30 (58.82)	21 (41.18)		
		Cancer	6 (100)	0 (0)		
7	Employment Status	Student	1923 (64.23)	1071 (35.77)	256.60	0.000
		Full-Time	531 (62.47)	319 (37.53)		
		Part-Time	156 (86.67)	24 (13.33)		
		Unemployed	72 (44.44)	90 (55.56)		
		Housewife	6 (5.26)	108 (94.74)		
		Retired	62 (83.78)	12 (16.22)		
		Worker	18 (100)	0 (0)		
8	Education	Graduate	1004 (58.44)	714 (41.56)	170.86	0.000
		College	1162 (75.60)	375 (24.40)		
		Postgraduate	590 (52.73)	529 (47.27)		
		High School	12 (66.67)	6 (33.33)		

**Table 4:** The intentions toward COVID-19 vaccines among General Populations (*n* = 4392).

Q.No.	Questions/Variables	Values	Frequency	Percentage
1	I feel safe after being vaccinated against COVID-19	Agree	1731	39.41
		Neutral	1462	33.29
		Strongly agree	947	21.56
		Disagree	174	3.96
		Strongly disagree	78	1.78
2	I can feel that my family is protected after getting vaccinated against COVID 19	Agree	1534	34.92
		Neutral	1462	33.29
		Strongly agree	892	20.31
		Disagree	432	9.84
		Strongly disagree	72	1.64
3	I can rely on COVID-19 vaccines to stop the pandemic	Neutral	1726	39.3
		Agree	1654	37.66
		Strongly agree	640	14.57
		Disagree	252	5.74
		Strongly disagree	120	2.73
4	Although most vaccines appear to be safe, there may be problems that we have not yet discovered	Agree	2225	50.66
		Neutral	1135	25.84
		Strongly agree	612	13.93
		Disagree	360	8.2
		Strongly disagree	60	1.37
5	COVID-19 vaccines can cause unforeseen problems in some population groups.	Agree	1671	38.05
		Neutral	1528	34.79
		Disagree	516	11.75
		Strongly disagree	383	8.72
		Strongly agree	294	6.69
6	I worry about the unknown effects of vaccines in the future.	Agree	1747	39.78
		Neutral	1377	31.35
		Disagree	684	15.57
		Strongly agree	354	8.06
		Strongly disagree	230	5.24
7	Authorities promote vaccination for financial gain, not for people’s health.	Neutral	1593	36.27
		Disagree	1064	24.23
		Agree	802	18.26
		Strongly disagree	741	16.87
		Strongly agree	192	4.37
8	COVID-19 vaccination programs are a big con	Agree	1488	33.88
		Neutral	1254	28.55
		Disagree	960	21.86
		Strongly disagree	486	11.07
		Strongly agree	204	4.64
9	I am worried about the rapid development of the COVID vaccine	Agree	1384	31.51
		Neutral	1334	30.37
		Disagree	1098	25
		Strongly disagree	336	7.66
		Strongly agree	240	5.46
10	I have had a prior reaction to other vaccines, and I am worried about a reaction to COVID vaccination	Disagree	1307	29.76
		Neutral	1251	28.49
		Agree	1044	23.77
		Strongly disagree	568	12.93
		Strongly agree	222	5.05
11	I do not get vaccinated because of a fear of needles	Disagree	1571	35.76
		Strongly disagree	1557	35.45
		Neutral	714	16.26
		Agree	490	11.16
		Strongly agree	60	1.37
12	Natural immunity lasts longer than COVID-19 vaccination.	Agree	1698	38.65
		Neutral	1372	31.24
		Disagree	674	15.35
		Strongly agree	486	11.07
		Strongly disagree	162	3.69
13	Natural exposure to novel corona gives the safest protection	Neutral	1615	36.77
		Disagree	1177	26.8
		Agree	1140	25.96
		Strongly agree	286	6.51
		Strongly disagree	174	3.96
14	Being exposed to diseases naturally is safer for the immune system than being exposed through vaccination	Agree	1288	29.33
		Neutral	1270	28.92
		Disagree	968	22.04
		Strongly agree	482	10.97
		Strongly disagree	384	8.74

**Table 5:** Association between intentions and demographic variables (*n* = 4392).

S.No.	Variable	Categories	Positive Frequency (%age)	Negative Frequency (%age)	Chi-Square	Significance (*p*-Value)
1	Gender	Male	2535 (93.78)	168 (6.22)	1.84	0.390
		Female	1563 (92.87)	120 (7.13)		
		Prefer not to say	6 (100)	0 (0)		
2	Age	18–29	3031 (92.49)	246 (7.51)	20.83	0.000
		30–49	999 (95.97)	42 (4.03)		
		Above 50	74 (100)	0 (0)		
3	Administrative Units	Khyber Pakhtunkhwa	1644 (90.13)	180 (9.87)	354.45	0.000
		Punjab	1170 (99.49)	6 (0.51)		
		Sindh	463 (100)	0 (0)		
		Islamabad Capital Territory	282 (75.81)	90 (24.19)		
		Baluchistan	268 (100)	0 (0)		
		Azad Jammu and Kashmir	150 (100)	0 (0)		
		Gilgit-Baltistan	127 (91.37)	12 (8.63)		
4	Residence	Urban	2620 (92.58)	210 (7.42)	9.67	0.0019
		Rural	1484 (95.01)	78 (4.99)		
5	Marital status	Single	3085 (92.12)	264 (7.88)	40.44	0.000
		Married	1019 (97.70)	24 (2.30)		
6	Chronic disease	No chronic disease	3295 (92.90)	252 (7.10)	106.01	0.000
		Cardiac disease	206 (100)	0 (0)		
		Hypertension	174 (100)	0 (0)		
		Overweight/obesity	156 (92.86)	12 (7.14)		
		Others	126 (95.45)	6 (4.55)		
		Asthma or respiratory disease	108 (100)	0 (0)		
		Kidney or liver disease	33 (64.71)	18 (35.29)		
		Cancer	6 (100)	0 (0)		
7	Employment Status	Student	2784 (92.99)	210 (7.01)	112.36	0.000
		Full-Time	838 (98.59)	12 (1.41)		
		Part-Time	150 (83.33)	30 (16.67)		
		Unemployed	132 (81.48)	30 (18.52)		
		Housewife	108 (94.74)	6 (5.26)		
		Retired	74 (100)	0 (0)		
		Worker	18 (100)	0 (0)		
8	Education	Graduate	1664 (96.86)	54 (3.14)	56.11	0.000
		College	1399 (91.02)	138 (8.98)		
		Postgraduate	1023 (91.42)	96 (8.58)		
		High School	18 (100)	0 (0)		

## Data Availability

On reasonable request, the corresponding author will provide the datasets used and analyzed in this study.
